# Restoration of Proper Trafficking to the Cell Surface for Membrane Proteins Harboring Cysteine Mutations

**DOI:** 10.1371/journal.pone.0047693

**Published:** 2012-10-17

**Authors:** Angelica Lopez-Rodriguez, Miguel Holmgren

**Affiliations:** Neurophysiology Section, Porter Neuroscience Research Center, National Institute of Neurological Disorders and Stroke (NINDS), National Institutes of Health (NIH), Bethesda, Maryland, United States of America; Purdue University, United States of America

## Abstract

A common phenotype for many genetic diseases is that the cell is unable to deliver full-length membrane proteins to the cell surface. For some forms of autism, hereditary spherocytosis and color blindness, the culprits are single point mutations to cysteine. We have studied two inheritable cysteine mutants of cyclic nucleotide-gated channels that produce achromatopsia, a common form of severe color blindness. By taking advantage of the reactivity of cysteine’s sulfhydryl group, we modified these mutants with chemical reagents that attach moieties with similar chemistries to the wild-type amino acids’ side chains. We show that these modifications restored proper delivery to the cell membrane. Once there, the channels exhibited normal functional properties. This strategy might provide a unique opportunity to assess the chemical nature of membrane protein traffic problems.

## Introduction

Improper targeting of membrane proteins causes many diseases. Often point mutations to cysteine hinder the delivery of membrane proteins to the cell surface [1], [2], [3], [4], [5], [6], [7], or to the correct side of polarized cells [8], [9]. Because cysteine is a readily reactive amino acid, in principle it should be possible to recover proper trafficking by modifying its chemical structure in order to mimic the side chain of the wild type amino acid. As a proof of principle, we have studied two naturally occurring cysteine mutations in a cyclic nucleotide-gated channel (CNGA3) responsible for hereditary cone photoreceptor disorders: Y181C linked to incomplete achromatopsia and R277C linked to complete and incomplete achromatopsia or cone dystrophy [10], [11]. We have chosen these mutations because proper surface CNG channel expression can be easily assayed using electrophysiological techniques, and because both mutations, which cause channel retention in the endoplasmic reticulum (ER) [11], [12], change wild type amino acids of drastically different chemistries.

**Figure 1:**
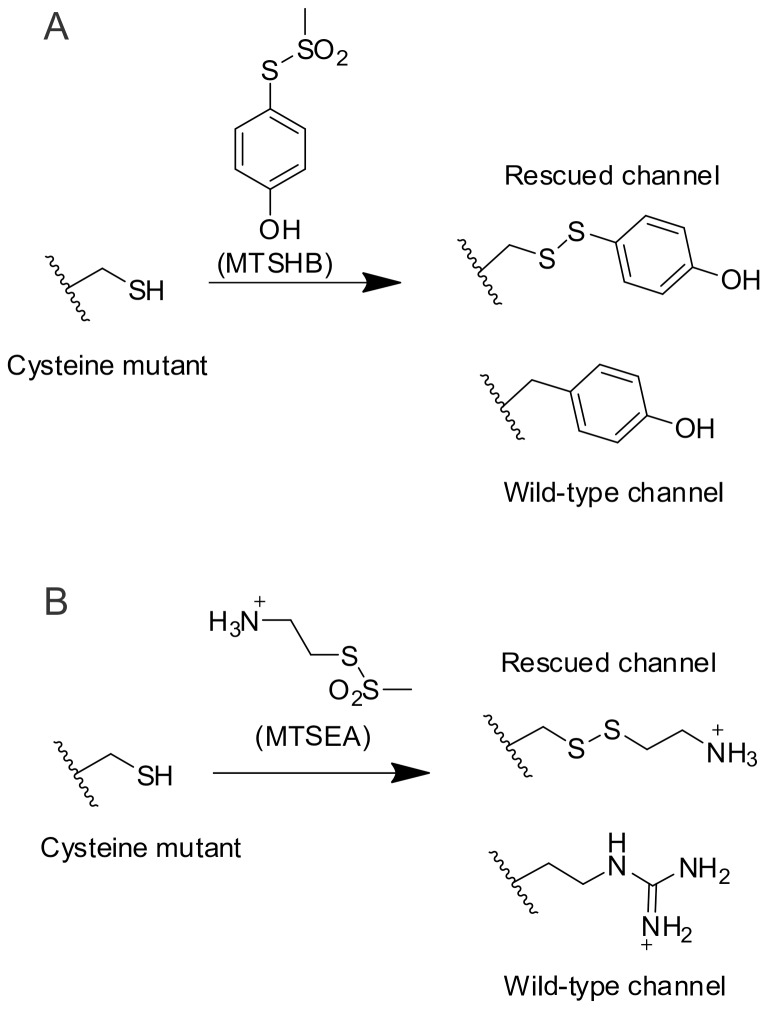
Rescuing strategy. A. Via a disulfide bridge, MTSHB attaches a hydroxyl benzene moiety that mimics tyrosine’s side chain. B. Via a disulfide bridge, MTSEA introduces a primary amine which mimics arginine’s side chain.

CNG channels open a cationic selective permeation pathway in response to intracellular cyclic nucleotides [13], [14]. In the visual system, CNG channels are key players in the transduction of light into electrical signals [15]. In native cells, these channels are formed by the coassembly of four homologous subunits [16], [17], [18], [19], [20], [21], each containing six transmembrane segments. Functional homotetramers can be formed by the CNGA1, A2 or A3 subunits [22], [23], [24], and these channels are usually studied as homotetramers in heterologous systems. We have introduced both achromatopsia-related cysteines in a cysteine-less CNGA1 channel [25], and used them as a target for specific chemical modification with hydroxybenzyl- (MTSHB) and aminoethyl-methanethiosulfonate (MTSEA). These reagents readily attach to the side chain of cysteines and mimic the chemistry of tyrosine and arginine, respectively (**Fig. 1**). Although Y181C and R277C caused ER retention, after chemical modification both mutants were targeted to the cell surface, providing a unique opportunity for their functional characterization.

## Materials and Methods

### Mutagenesis and Expression

cDNA of a cysteine-less CNGA1 channel was kindly provided by William Zagotta (University of Washington, Seattle, WA). Cysteine mutations were introduced in this background using a QuickChange kit (Stratagene). Amino acid substitutions, as well as the integrity of the entire coding region of each channel, were confirmed by DNA sequencing (NINDS sequencing facility). A CNGA1-Green Fluorescent Protein (CNG-GFP) was created with standard PCR techniques. cRNAs were synthesized with a T7 promoter-based *in vitro* transcription protocol (Ambion). *Xenopus* oocytes were injected with 50 nl (500 ng/µl) of cRNA and incubated in ND96 solution (in mM: 96 NaCl, 2 KCl, 1 MgCl_2_, 1.8 CaCl_2_, 5 HEPES, pH 7.6) at17°C for two to three days to allow channel to express. To assess surface expression by fluorescence, we engineered two cysteine-less background constructs: CNGA1-GFP and CNGA1-FLAG. GFP and the FLAG epitope (DYKDDDDK) were inserted in frame immediately before the stop codon. In general, CNG channels tolerate these tags at the carboxy-terminal remarkably well [1], [18], [19]. This study was approved by National Institute Neurological Disorders and Stroke/National Institute on Deafness and Other Communication Disorders Animal Care and Use Committee Protocol Number 1253-09.

### Chemical Treatment

R272C mutant channels were rescued using aminoethyl-methanethiosulfonate (MTSEA), a compound which readily permeates the membrane of cells, including that of *Xenopus* oocytes, in its uncharged form [26]. Treatments were performed 48 hrs after cRNA injection. MTSEA (final concentration 2 mM) was prepared in ND96 solution and added into wells containing oocytes. Incubation was performed at 17°C for six hours. Fresh ND96 solution with MTSEA was replaced every 30 minutes. These prolonged treatments were not readily tolerated by all oocytes. We found that after around 4 hours of MTSEA exposure, about 50% of the oocytes began to show signs of deterioration, as the animal pole became pale. Those oocytes were not used for any experiments.

Y176C mutant channels were treated with hydroxybenzyl-methanethiosulfonate (MTSHB). Oocytes were injected once with 50 nl of a 40 mM MTSHB stock solution, which was prepared in an ethanol-DMSO mix (50/50), and incubated at 17°C overnight.

After each treatment, some oocytes were used to analyze the extent of protein trafficking by fluorescence or immunocytochemistry, membrane protein expression assays, and others were used for electrophysiological characterization.

### Biotinylation of Membrane Surface Protein

Six to eight oocytes were incubated for 1 hr at 4°C in ND96, supplemented with 50 µg/ml gentamicin and 1.0 mg/ml sulfo-NHS-LC-biotin (Pierce). Then, oocytes were washed several times with ND96 supplemented with 100 mM glycine and lysed in 200 µl of buffer H (1% Triton X-100, 100 mM NaCl, 20 mM Tris-HCl, pH 7.4 with protease inhibitors (SIGMA) by trituration. Lysates were rocked at room temperature for 15 min and then centrifuged at 13,000 rpm for 3 min. The pellet was discarded and the supernatant was divided in two equal samples, one containing total proteins and the other to be used for preparation of cell membrane proteins. For cell membrane protein isolation, 50 µl of NeutrAvidinTM Agarose Resin (Thermo Scientific) was added to the sample and rocked gently at 4°C for at least 30 min. Resin was washed at least 6 times with buffer H, and finally eluted in buffer H (supplemented with 10% 2-ME and 50 mM DTT) to the same volume as the sample containing total proteins, and incubated for 5 min at 95°C.

All samples were deglycosylated using PNGase F (New England BioLabs) before being loaded into a SDS-PAGE gel and transferred to a polivinylidene difuoride (PVDF) membrane. Blots were probed with an anti-GFP mouse monoclonal antibody at a dilution of 1∶5000 (Clontech). Primary antibodies were detected with a secondary, goat anti-mouse antibody conjugated to horseradish peroxidase used at a dilution of 1∶10000 (Pierce). Membranes were developed by SuperSignal WestFemto (Thermo Scientific) and visualized by chemioluminiscence using a FluorChem E Imager (Cell Biosciences). Analysis was performed using Image AlphaView software (Cell Biosciences).

### Immunofluorescent Staining

The oocyte’s vitelline layer was removed to reduce background fluorescence. Oocytes were permeabilized with 0.03% saponin in ND96 solution for 20 min, washed with ND96 solution, blocked with 1% BSA in ND96 for 30 min. and incubated overnight with a 1∶250 dilution of a FLAG polyclonal antibody (Santa Cruz Biotechnology. Inc.) in ND96 at 4°C. Oocytes were washed several times with ND96 and incubated for 1 hr in a 1∶500 dilution of secondary antibody (Texas Red conjugated donkey anti-goat IgG; Santa Cruz Biotechnology. Inc.) at 4°C. After several rinses with ND96, oocytes were imaged using a Zeiss LSM 510 confocal microscope.

### Electrophysiology

The recording solution consisted of (in mM): 120 NaCl, 2 EDTA, 10 HEPES (pH = 7.4). All reagents were obtained from SIGMA. Currents from inside-out excised patches [27] were acquired using an Axopatch 200B amplifier (Molecular Devices) and a Digidata 1322 acquisition board (Molecular Devices), and were sampled between 2.5 to 10 kHz using a low-pass filter at 1 or 2 kHz. Patch electrodes with tip diameters between 8 and 15 µm were made with borosilicate glass pipettes. Macroscopic data analysis was performed with pClamp 9 (Molecular Devices) and Origin 8 (Microcal Software) software. Solutions were changed using a computer-controlled rapid solution changer (RSC-200; Biologic Science Instruments).

## Results

### Recovering Cell Membrane Expression of CNG Channels Containing the R272C Mutation

To assess whether chemical modification could restore cell surface expression and functionality, we used the well-characterized bovine CNGA1 channel which lacks all native cysteines [28]. Position R272 in the bovine CNGA1 channel is equivalent to position R277 in the human CNGA3 channel and is located within a large domain known as the voltage sensor. From the crystal structure of a mammalian voltage-activated potassium (K_V_) channels [29], a cousin of CNG channels, this position is part of the fourth transmembrane segment (S4) where the critical charges that sense the transmembrane voltage are spaced every three amino acids [30], [31], [32]. Little is known about the role of the voltage sensor in CNG channels due to technical difficulties, such as poor surface expression of channels harboring S4 mutations [4], [12]. In all channels within the superfamily of voltage-activated ion channels, which includes CNG channels, it is known that S4 is important for maturation [4], [12], [33], [34], [35].

**Figure 2:**
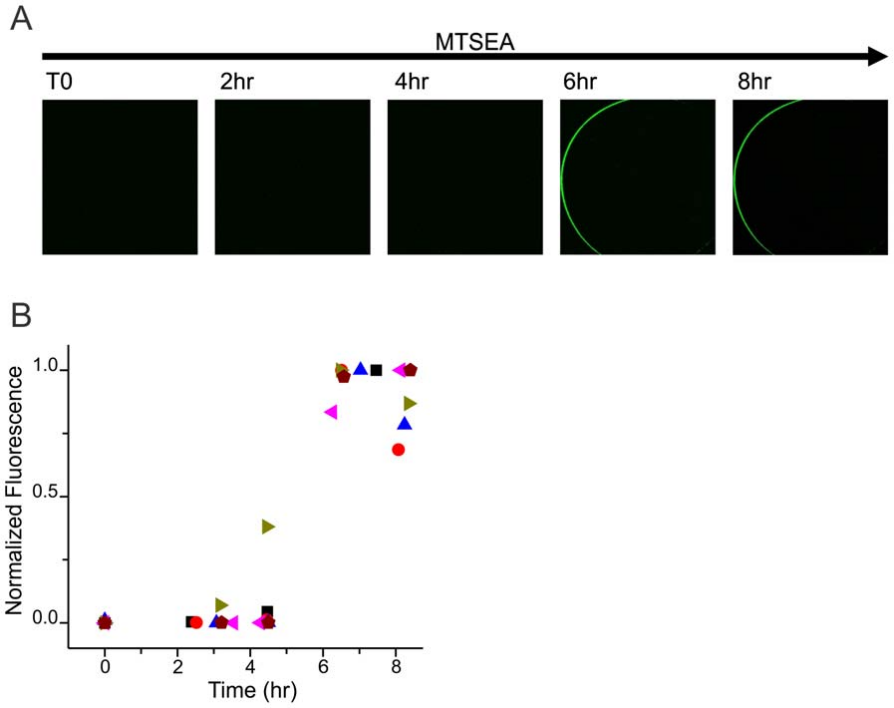
Time course of cell surface expression for R272C CNGA1-GFP channels exposed to MTSEA. A. Representative confocal images of one oocyte before (T0) and after 2 mM MTSEA exposure. Media with fresh MTSEA was exchanged every 30 min. After ∼6 hrs of MTSEA treatment, GFP fluorescence was detectable at the oocyte’s surface. B. Plot shows the time course of cell surface fluorescence detection in six different oocytes.

To verify that the R272C mutation impedes surface expression in the cysteine-less CNGA1-GFP background, we injected this construct into *Xenopus* oocytes. After two days, we could not observe channels at the cell surface using fluorescence microscopy. Further, using functional expression as an indicator, we were unsuccessful in detecting cGMP-activated ionic currents in more than 40 excised inside-out membrane patches (not shown). However, by incubating injected oocytes with a solution containing 2 mM MTSEA (a membrane permeant reagent [26] that leaves a moiety mimicking the side chain of arginine, as shown in Fig. 1) we restored the proper channel trafficking. Fig. 2A shows confocal images of an oocyte in which R272C CNGA1-GFP channels were rescued after ∼6 hr of MTSEA treatment. Fig. 2B shows the time course of fluorescence detection at the cell surface of six oocytes. A similar MTSEA treatment to cysteine-less CNGA1-GFP channels has no effect on cell surface expression (Fig. S1).

**Figure 3:**
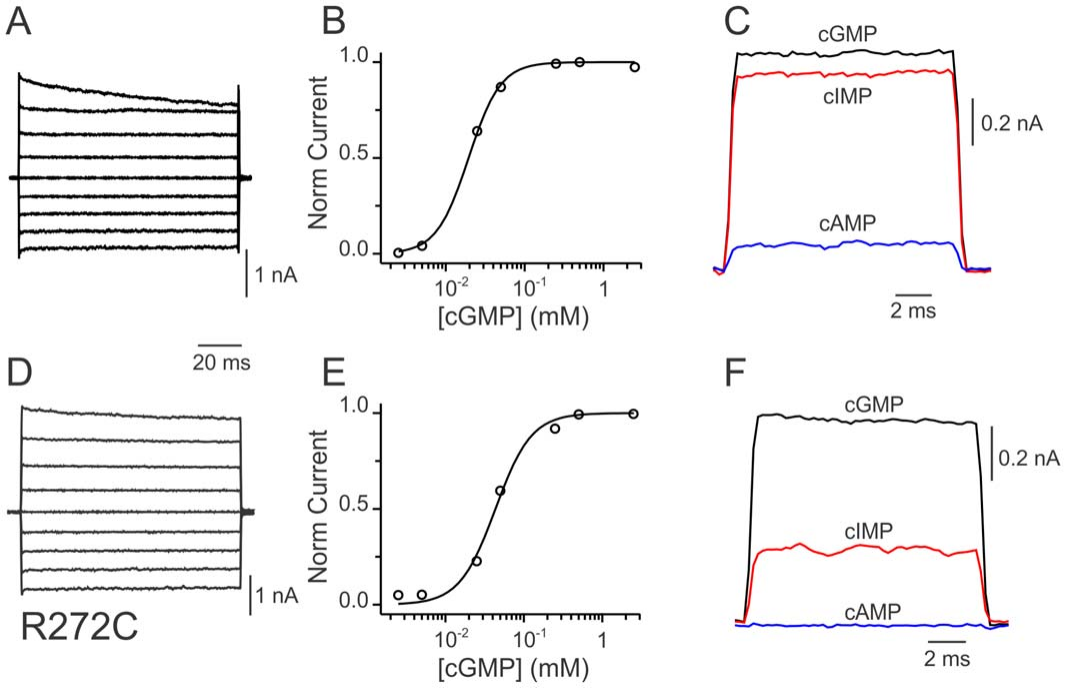
Functional characterization of CNGA1-GFP channels at the cell surface. A, B & C. Cysteine-less CNGA1-GFP channels. A, Ionic currents in the presence of saturating [cGMP] (2 mM). cGMP-activated currents shown were acquired in response to 100 ms voltage steps from −80 to +80 mV (every 20 mV) from a holding potential of 0 mV. B. Dose response for cGMP at +60 mV. Solid line represents a normalized Hill equation fit to the data. The best fit parameter values for K½ was 19±1 µM, and for the hill coefficient (n) was 2.1±0.2. Average values for K½ and n were 24±3 µM and 2.16±0.46, respectively (n = 4 oocytes). C. Efficacies for different agonists. Wild type CNGA1 channels displayed larger than 95% maximal probability of opening with saturated concentrations of cGMP, less with cIMP, and much less with cAMP. Nucleotide-activated current records shown were obtained from the same excised inside out patch with saturating concentrations of cGMP (2 mM; *black*), cIMP (16 mM; *red*) and cAMP (16 mM; *blue*). D, E & F. Rescued R272C CNGA1-GFP channels. D. Ionic current carried by MTSEA-modified R272C channels. In response to a comparable experimental protocol as in A, rescued channels were able to conduct ionic current with similar properties as wild type channels. E. Dose response of modified R272C channels at the cell surface for cGMP. Solid line corresponds to a normalized Hill equation fit to the data. The best fit parameter values for K½ and n were 43.4±2.5 µM and 1.9±0.3, respectively. Average values for K½ and n were 59±10 µM and 1.48±0.22, respectively (n = 14 oocytes). F. Agonist efficacies for rescued R272C mutant channels. Nucleotide-activated current records shown were acquired from the same patch, using the equivalent agonist concentration as in C. Similar observations were made in 3 different patches.

Modified channels respond normally to the presence of agonists. Fig. 3A shows cGMP-activated currents carried by control MTSEA-treated cysteine-less CNGA1-GFP channels in response to voltage steps between −80 and +80 mV from a holding potential of 0 mV in the presence of saturating [cGMP]. Under similar conditions, ionic currents from MTSEA-modified R272C mutant channels are comparable (Fig. 3D). Dose-response curves for cGMP at +60 mV for wild-type channels (Fig. 3B) and modified R272C channels (Fig. 3E) were also similar. Solid lines represent Hill equation fits in which K½ and n values were 19±1 µM and 2.2±0.2 for wild-type channels and 43±0.5 µM and 1.9±0.3 for modified 272C channels. Another property of CNGA1 channels is their sensitivity to saturated concentrations of the various cyclic nucleotide agonists. In general they barely open with cAMP, open more with cIMP and open with a high probability with cGMP. Both, cysteine-less CNGA1-GFP (Fig. 3C) and modified R272C (Fig. 3F) channels maintained the same relative efficacy among these agonists. Taken together, these results demonstrate that attaching a primary amine moiety to cysteine 272 can successfully mimic the role played by the side chain of arginine in wild-type channels. Specifically, this modification restores targeting to the cell surface and produces channels that function relatively normal.

**Figure 4:**
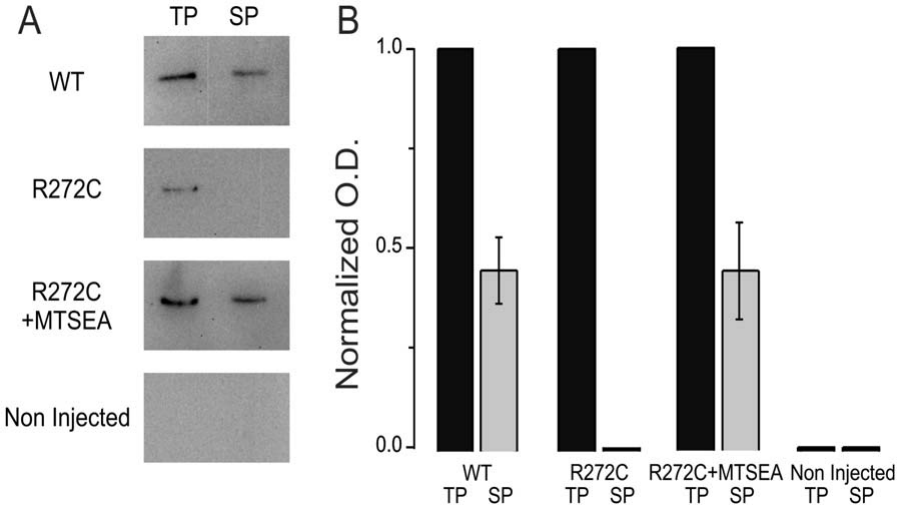
Cell surface expression of CNGA1-GFP channels. A. Representative Western blot of total CNGA1-GFP protein (TP) and biotinylated CNGA1-GFP cell surface protein (SP). WT, R272C, R272C+MTSEA denote cysteine-less CNGA1-GFP, mutant channels that were not treated with MTSEA and mutant channels that were modified by MTSEA, respectively. An expected band of ∼106 kDa for the deglycosylated wild-type GFP tagged channel was detected by chemiluminiscence using a GFP antibody. No signal was detected in non injected oocytes. B. Densitometry analysis of the bands normalized to TP (n = 3 different oocytes batches).

How efficiently can a MTSEA-modified R272C CNGA1-GFP channel be rescued? To approach this question, we assessed total and cell surface protein expression from pools of six to eight oocytes (see Methods). For cysteine-less CNGA1-GFP channels, ∼45% of the total membrane protein is at the cell surface (Fig. 4; WT). Untreated R272C channels cannot be detected at the cell surface (Fig. 4; R272C), consistent with previous observations [4], [12]. However, treatment with MTSEA successfully rescued R272C CNGA1 channels to the cell surface, at comparable proportions as cysteine-less CNGA1-GFP channels (Fig. 4; R272C+MTSEA). These results suggest that the added moiety to cysteine 272 restored the proper channel conformation allowing it to pass the various trafficking checkpoints.

**Figure 5:**
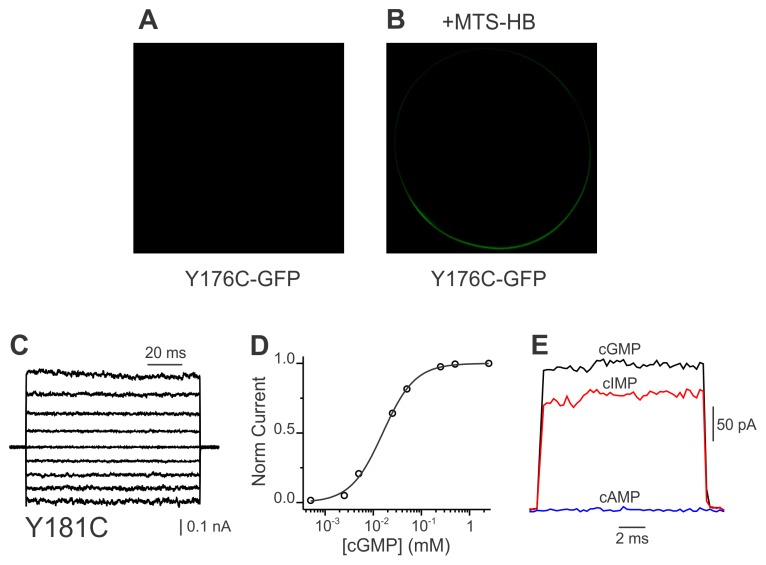
Y176C CNGA1-GFP mutant channels. A. Confocal image of an oocyte injected with cRNA encoding for Y176C CNGA1-GFP channels. We never detected any signs of proper trafficking in >100 oocytes by standard fluorescence microscopy, suggesting that these channels do not reach the cell surface. B. MTSHB treatment recovers proper trafficking of Y176C mutant channels to the cell surface. Confocal image shows GFP fluorescent signal at the cell surface of an oocyte expressing Y176C mutant channels. MTSHB was injected ∼ 12 h before acquiring the image (n >25 oocytes). C. Ionic currents in the presence of a saturating cGMP concentration (2 mM), in response to the same voltage protocol as described in Fig. 3A. D. Dose response for cGMP at +60 mV. Solid line represents a normalized Hill equation fit to the data. The best fit parameter values for K½ and n were 16±1 µM and 1.30±0.05, respectively. From a total of 8 oocytes, the average values for K½ and n were 16±3 µM and 1.6±0.1, respectively. E. Agonist efficacies of rescued Y176C mutant channels. Nucleotide-activated current records shown in the presence of saturating concentrations of each agonist (same as Fig. 3C) were acquired from the same excised patch. Rescued Y176C channels showed a similar efficacy pattern as cysteine-less CNGA1-GFP channels. Similar observations were observed in 4 patches.

Our approach was also successful with other positively charged residues. Unlike the case for K_V_ channels, the substitution of cysteine for the positively charged, voltage sensing amino acids within the S4 transmembrane segment of CNG channels leads to immature products that get trapped in the ER [12]. We tested the ability of MTSEA to rescue surface expression of R269C, R275C and R278C. In each case, mutants were efficiently redirected to the cell membrane, demonstrating the general applicability of the technique (Fig. S2).

### Restoring Proper Targeting to Y176C Mutant CNG Channels

Position Y176 in CNGA1 channels corresponds to position Y181 in hCNGA3 channels and is located within the first transmembrane segment. As in hCNGA3 channels [11], the Y176C mutation impeded Y176C CNGA1-GFP channels from reaching the cell membrane (Fig. 5A). Chemical modification with MTSHB would attach a moiety to these cysteines that resembles the original tyrosine side chain (Fig. 1A). This reagent, however, is very hydrophobic and precipitates in aqueous solutions. We were unable to find an experimental condition where we could incubate oocytes in MTSHB to restore function. However, by directly injecting a 40 mM MTSHB solution into the oocytes, we were able to recover cell surface expression (Fig. 5B). Likely, the rather oily environment of an oocyte’s yolk allowed the reagent to stay in solution at sufficiently high concentrations to modify Y176C. A direct reagent injection has been successfully used before to probe proton channel function in a tryptophan mutant [36]. Modified Y176C CNGA1-GFP channels responded normally to saturating concentrations of cGMP (Fig. 5C), although current levels were consistently smaller than cysteine-less and rescued R272C CNGA1 channels. Likely, a single MTSHB injection (c.f. freshly applied MTSEA every 30 min for R272C CNGA1 channels) and the relatively fast hydrolysis of MTS reagents contributed to the lower levels of rescued Y176C CNGA1 channels. The dose-response for cGMP at +60 mV for modified Y176C CNGA1-GFP channels was characteristic of CNGA1 channels (Fig. 5D). The solid line through the data corresponds to a Hill equation fit with K½ and n values of 16±1 µM and 1.3±0.05. Finally, rescued channels showed a similar efficacy for saturating concentrations of different agonists (Fig. 5E) as for the cysteine-less CNGA1-GFP channels (Fig. 3E). These studies demonstrate that Y176C mutant channels retained in intracellular compartments can be successfully targeted to the cell surface by adding a side-chain to a cysteine that mimics that of tyrosine. Once at the cell surface, these channels behave normally.

## Discussion

We describe a method to restore proper maturation and trafficking of membrane proteins that have been retained within intracellular organelles due to single point mutations to cysteine. Because the side chain of cysteine is highly reactive, we reasoned that modification with reagents that restored the original chemistry could drive proper maturation. We successfully restored both trafficking and normal function to CNGA1 mutant channels R272C and Y176C, both responsible for hereditary cone photoreceptor disorders [10], [11].

Maturation of any protein is a complex, multi-step process involving a network of intracellular proteins and organelles. Surely, all genetic mutations leading to defective maturation cannot be repaired by a single strategy. Thus far, a variety of experimental approaches have been shown to recover proper maturation. For example, cell surface expression of mutant HERG and CNGA3 channels [6], [37], as well as deficient lysosomal glucocerebrosidase [38], can be restored simply by lowering the temperature of incubation. Based on their ability to stabilize proper folding conformations in the ER, drugs and lipid chaperones are emerging as new strategies to restore protein maturation [39], [40], [41], [42], [43]. As with other rescue methods, ours has disadvantages: it is restricted to cysteine mutants and it is not specific since MTS reagents will modify any accessible cysteine in a protein. Nevertheless, outside of therapeutics, this method has applications that could, in principle, offer relevant structural and functional information about diseases. For example, it could be used to better understand the chemical nature of the protein folding failure since many different MTS reagents are available that attach moieties resembling different amino acid side chains. Another potential use could be for kinetic studies of folding. For example, if a cysteine mutation impairs proper folding, modification reactions by MTS reagents are sufficiently fast to permit the temporal resolution of downstream conformational changes, providing kinetic information on folding steps. A third application we envision is to use the disease related mutant in the same way that biophysicists use engineered cysteines as a tool to study state dependent accessibility. This will provide information of conformational changes at the site of the cysteine mutation.

## Supporting Information

Figure S1 **Cell surface expression time course of cysteine-less CNGA1-GFP channels exposed to MTSEA.** A. Representative confocal images of one oocyte before (T0) and after 2 mM MTSEA exposure. Media with fresh MTSEA was exchanged every 30 min. MTSEA treatment does not affect cell surface expression of “wild-type” channels.
(TIF)

Figure S2 **Cell surface targeting of S4 cysteine mutants by chemical modification.** All images shown were obtained after immunocytochemical labeling of oocytes expressing CNGA1 arginine to cysteine mutations in the S4 transmembrane segment. The absence of fluorescence at the oocytes’ cell membrane (left column) indicate that these arginine to cysteine mutations in the S4 segment render immature channels that are unable to reach the cell surface. After 6 h MTSEA treatment, we were able to restore proper trafficking to these mutant channels (right column). Representative of >10 cells in each panel.
(TIF)
