# Supplementary figures and images for: Restoration of Proper Trafficking to the Cell Surface for Membrane Proteins Harboring Cysteine Mutations
Source: PLoS One. 2012 Oct 17;7(10):e47693. doi: 10.1371/journal.pone.0047693 (PMC3474720; doi:10.1371/journal.pone.0047693)

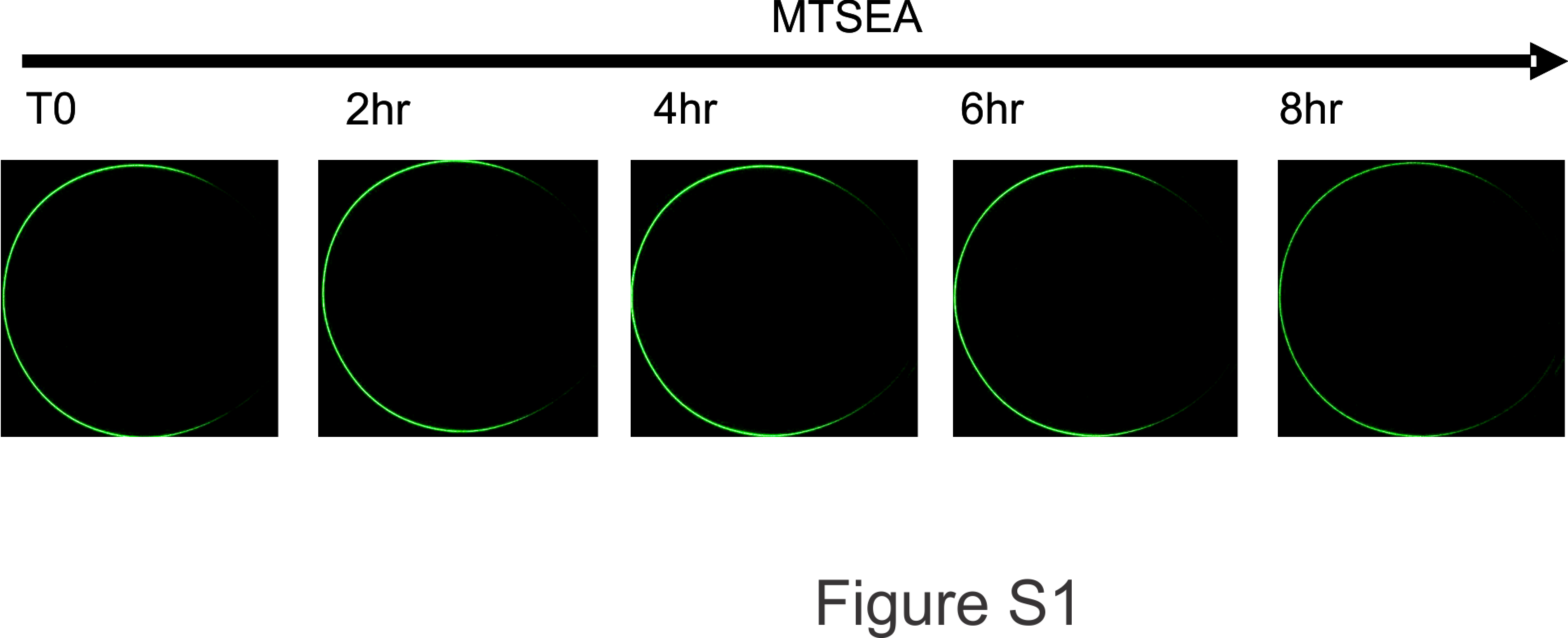

Supplement: Figure S1 — Cell surface expression time course of cysteine-less CNGA1-GFP channels exposed to MTSEA. A. Representative confocal images of one oocyte before (T0) and after 2 mM MTSEA exposure. Media with fresh MTSEA was exchanged every 30 min. MTSEA treatment does not affect cell surface expression of “wild-type” channels. (TIF) [file pone.0047693.s001.tif]

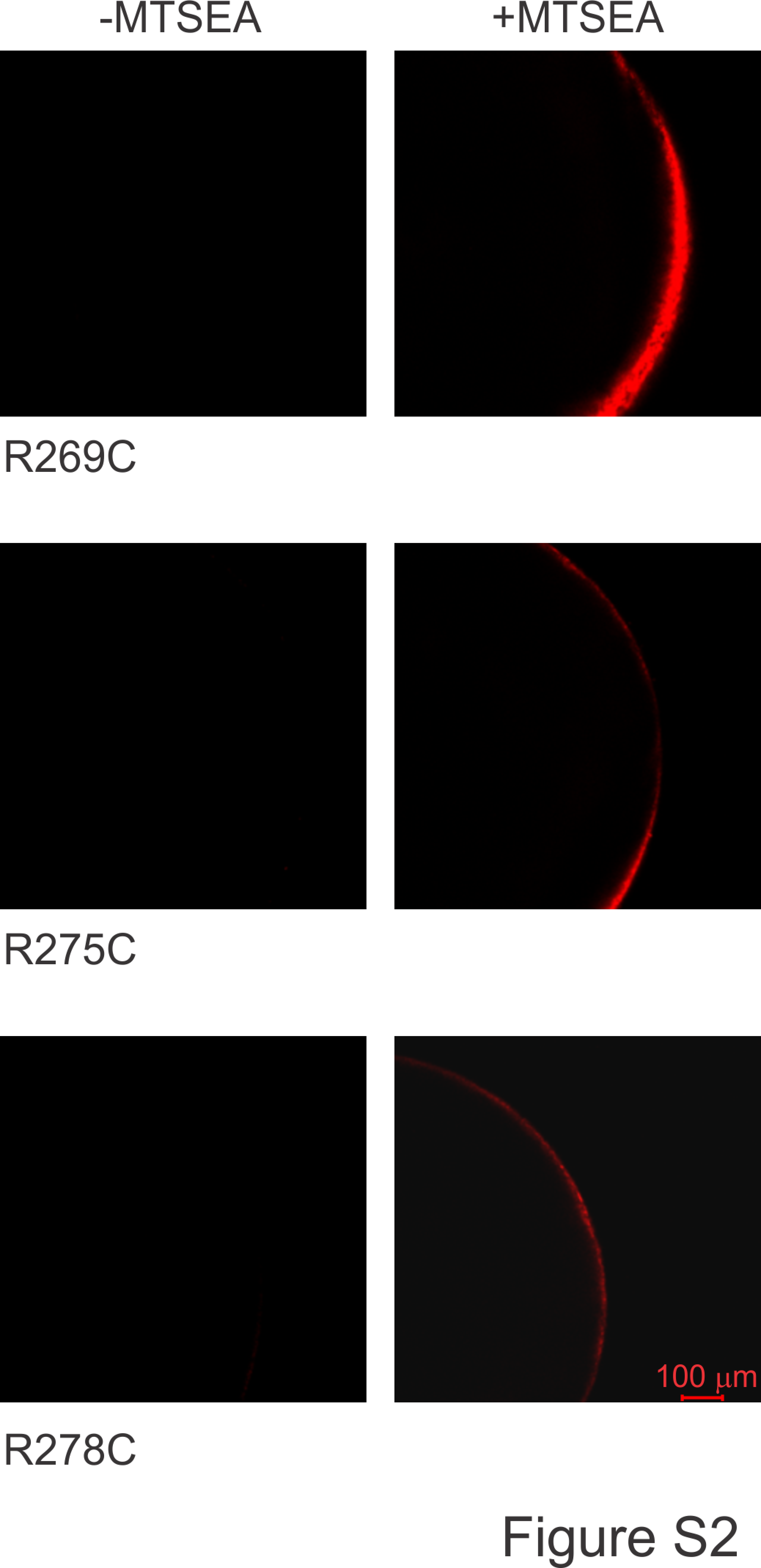

Supplement: Figure S2 — Cell surface targeting of S4 cysteine mutants by chemical modification. All images shown were obtained after immunocytochemical labeling of oocytes expressing CNGA1 arginine to cysteine mutations in the S4 transmembrane segment. The absence of fluorescence at the oocytes’ cell membrane (left column) indicate that these arginine to cysteine mutations in the S4 segment render immature channels that are unable to reach the cell surface. After 6 h MTSEA treatment, we were able to restore proper trafficking to these mutant channels (right column). Representative of >10 cells in each panel. (TIF) [file pone.0047693.s002.tif]
